# Validation of grip strength as a measure of frailty in rheumatoid arthritis

**DOI:** 10.1038/s41598-022-21533-5

**Published:** 2022-12-06

**Authors:** Yasumori Sobue, Mochihito Suzuki, Yoshifumi Ohashi, Hiroshi Koshima, Nobuyuki Okui, Koji Funahashi, Hisato Ishikawa, Hidenori Inoue, Masayo Kojima, Shuji Asai, Kenya Terabe, Kenji Kishimoto, Masataka Maeda, Daisuke Kihira, Shiro Imagama, Toshihisa Kojima

**Affiliations:** 1 Department of Orthopedic Surgery, Japanese Red Cross Nagoya Daiichi Hospital, 3-35 Michishita, Nakamura, Nagoya, Aichi 453-8511 Japan; 2 Department of Orthopedic Surgery, Nagoya University Graduate School of Medicine, 65 Tsurumai, Showa, Nagoya, Aichi 466-8550 Japan; 3 Department of Orthopedic Surgery, Japan Community Health Care Organization Kani Tono Hospital, 1221-5 Dota, Kani, Gifu, 509-0206 Japan; 4 Department of Orthopedic Surgery, Yokkaichi Municipal Hospital, 2-2-37 Shibata, Yokkaichi, Mie 510-8567 Japan; 5 Department of Orthopedic Surgery, Kariya Toyota General Hospital, 5-15 Sumiyoshi, Kariya, Aichi 448-0852 Japan; 6 Department of Frailty Research, Center for Gerontology and Social Science, National Center for Geriatrics and Gerontology, 7-430 Morioka, Obu, Aichi 474-8511 Japan; 7 Department of Orthopedic Surgery, National Hospital Organization Nagoya Medical Center, 4-1-1 Sannomaru, Naka, Nagoya, Aichi 460-0001 Japan

**Keywords:** Health care, Rheumatology, Signs and symptoms

## Abstract

Rheumatoid arthritis (RA) patients often exhibit finger/wrist joint symptoms and reduced grip strength. This study aimed to validate grip strength as a measure of frailty in RA patients. Subjects were 424 female RA patients (mean age ± standard deviation, 66.8 ± 14.5 years). Frailty was defined as a score of ≥ 8 points on the Kihon Checklist (KCL). Finger/wrist joint symptoms were defined based on tender or swollen joints. Associations between frailty and grip strength were determined using receiver operating characteristic (ROC) curve analysis and multivariable logistic regression analysis. There were 179 subjects with frailty (42.2%). Multivariable logistic regression analysis revealed that frailty was significantly associated with grip strength independently of finger/wrist joint symptoms. In ROC curves, cut-off scores of grip strength for frailty in subjects without and with finger/wrist joint symptoms were 17 kg (sensitivity, 62.1%; specificity, 69.0%) and 14 kg (sensitivity, 63.2%; specificity, 73.0%), respectively. The results of the present study suggest that grip strength in female RA patients is associated with frailty, with a cut-off score of 17 kg (equivalent to Cardiovascular Health Study criteria, < 18 kg) when RA patients have no finger/wrist joint symptoms. However, when RA patients have finger/wrist joint symptoms, it may be considered to reduce the cut-off score of grip strength.

## Introduction

In a rapidly advancing aging society, a general goal for reducing nursing costs and improving quality of life is to maintain and improve physical function^1,2^. Frailty is a condition that leads to an increased need for long-term care and defined as the intermediate stages of health and disability with age-related declines in physical and cognitive function^3^. Various negative health outcomes, including COVID-19-related mortality are significantly associated with frailty^4,5^. Frailty can reduce healthy life expectancy, however, it can also be recovered with proper interventions since it is clinically considered as the condition of predisability^3^.

Rheumatoid arthritis (RA) is a chronic systemic inflammatory autoimmune disease which can lead to joint destruction and physical dysfunction due to synovial inflammation, as well as physical frailty^6^. The proportion of RA patients with frailty is estimated to be higher than that of the general population^7^, and the aging of RA patients is progressing^8,9^. As RA treatment has advanced significantly with the introduction of methotrexate, biologics, and Janus kinases inhibitors, the survival rate of RA patients has also improved in tandem^10^. In an aging society, especially in one like that of Japan, the extension of health life expectancy is important also for RA patients.

Grip strength reflects upper limb function as well as whole body physical function^11^. It has been reported that a decline in grip strength was associated with mortality, and monitoring grip strength may improve the identification of women at greatest risk of death^12^. Decreased function and muscle weakness have also been evaluated by grip strength in cancer patients^13^. Reduced grip strength is one of the Cardiovascular Health Study (CHS) criteria for diagnosing frailty^3,14^. A major benefit of grip strength is that it is easy to measure in clinical settings.

Findings from previous studies have highlighted the importance of detecting frailty early and preventing it in order to promote good health outcomes. However, RA patients often exhibit finger/wrist joint symptoms and reduced grip strength. Accordingly, this study aimed to validate the use of grip strength as a measure of frailty in RA patients, taking into consideration disease activity, especially finger/wrist joint symptoms.

## Materials and methods

### Subjects

This study included the RA patients who consecutively visited Japanese Red Cross Nagoya Daiichi Hospital, Japan Community Health Care Organization Kani Tono Hospital, and Yokkaichi Municipal Hospital between June and August 2021. All patients fulfilled the 2010 American College of Rheumatology (ACR)/European League Against Rheumatism (EULAR) classification criteria^15^. This study targeted only female RA patients. Cut-off scores of CHS criteria for males and females differ (i.e., < 18 kg for females and < 28 kg for males)^3,14^. Since this study aimed to confirm the validity of the cut-off score of grip strength, we first targeted females given the small number of male patients in the source population. Comorbidities were defined as diseases that were currently or previously treated by other medical specialists and included diabetes mellitus, hypertension, osteoporosis, interstitial pneumonia, and ever or current malignancy.

This retrospective study was approved by the Ethics Committees of Nagoya University School of Medicine (2017-0271), Japanese Red Cross Nagoya Daiichi Hospital (2020-451), Japan Community Health Care Organization Kani Tono Hospital (20110901), and Yokkaichi Municipal Hospital (2017-29). We disclosed information pertaining to the study at the cooperating facilities according to the procedure stipulated by the respective Ethics Committees. Informed consent was obtained from all subjects. The study was conducted in accordance with the World Medical Association of Helsinki ethical principles for medical research involving human subjects. Patients’ individual information was anonymized.

### Frailty

Frailty categories were defined based on Kihon Checklist (KCL) scores (≥ 8 points corresponds to frailty; 4–7 points corresponds to pre-frailty; 0–3 points corresponds to normal^16^), which is a widely used instrument developed by the Ministry of Health, Labour and Welfare in Japan to identify older people at risk of requiring care/support^17^. KCL consists of 25 self-reporting yes/no questions in total, including seven domains: activities of daily living, physical strength, nutrition, oral function, isolation, cognitive function, and depressive mood^16,17^. KCL was reported to be significantly correlated with Fried’s Cardiovascular Health Study criteria, and its validity as a screening tool to assess frailty has been demonstrated^3,16^.

### Grip strength

Grip strength was measured with the elbow fully extended in the standing position, using Smedley spring handgrip dynamometers (TTM Smedley Dynamo Meter; Tsutsumi, Tokyo, Japan). Measurements were carried out twice with the right hand and the left hand respectively, and the higher value was used for analysis, according to Asian Working Group for Sarcopenia^18^.

### Finger/wrist joint symptoms

First, we defined the dominant side as the higher value of grip strength. Then, finger/wrist joint symptoms were defined as tender and/or swollen joints on the dominant side. Tender and swollen joints referred to articular and periarticular manifestations including joint tenderness and swelling to palpation respectively^19^. Tender and swollen 28-joint count meant the number of tender and swollen joints out of 28 joints (knee, shoulder, elbow, wrist, metacarpophalangeal, proximal interphalangeal joints)^20^, and were incorporated into Disease Activity Score 28- C-reactive protein (DAS28-CRP): DAS28-CRP = 0.56*√(tender 28-joint count) + 0.28*√(swollen 28-joint count) + 0.014*(subject’s global assessment of disease activity visual analog scale) + 0.36*ln(CRP + 1) + 0.96^21^. DAS28-CRP was categorized as follows: clinical remission (DAS28-CRP < 2.3); low disease activity (LDA; 2.3 ≤ DAS28-CRP < 2.7); moderate disease activity (MDA; 2.7 ≤ DAS28-CRP ≤ 4.1); and high disease activity (HDA; DAS28-CRP > 4.1)^22,23^.

### Health Assessment Questionnaire-Disability Index (HAQ-DI)

HAQ-DI is 20 self-reporting questions consisting of eight categories (Dressing and grooming, Arising, Eating, Walking, Hygiene, Reaching, Gripping, and Other activities), and widely used as an index of physical function in RA patients^24^. HAQ-DI is calculated as the average of the highest score of questions in each category, and response coding scores of questions are as follows: “without any difficulty” = 0, “with some difficulty” = 1, “with much difficulty” = 2, and “unable to do” = 3^24^. HAQ-DI ≤ 0.5 is defined as functional remission^25^.

### Statistical analysis

Continuous variables are expressed as mean and standard deviation (SD) and were analyzed using the unpaired *t*-test. Ordinal variables and categorical variables are expressed as percentages and were analyzed using Fisher’s exact test. For the purpose of clarifying the associations between HAQ-DI and grip strength, and DAS28-CRP and finger/wrist joint symptoms, linear regression analyses adjusted for age and duration of disease were performed. Multivariable logistic regression analyses were performed to confirm the independent impact of related variables (e.g., grip strength) on frailty. Receiver operating characteristic (ROC) curves were generated to assess the associations between frailty, clinical remission (DAS28-CRP < 2.3)^23^, functional remission (HAQ-DI ≤ 0.5)^25^, and grip strength with or without finger/wrist joint symptoms. The point closest to the upper left corner was identified as the best cut-off point. Univariate analysis of variance was performed, and estimated marginal means of grip strength were calculated with age, duration of disease, and BMI as covariates, and stratifying by frailty and finger/wrist joint symptoms.

Statistical analyses were performed with EZR (Saitama Medical Center, Jichi Medical University, Saitama, Japan; http://www.jichi.ac.jp/saitama-sct/SaitamaHP.files/statmed.html), a graphical user interface for R (The R Foundation for Statistical Computing, Vienna, Austria)^26^, and SPSS version 28.0.0 software (IBM Corp., Armonk, NY, USA). P < 0.05 was considered statistically significant.

## Results

A total of 630 RA patients consecutively visited our hospitals between June and August 2021. Among these patients, data on clinical characteristics, including scores for grip strength, KCL, and DAS28-CRP were available for 591. Among these 591 patients, 424 were female. Subject characteristics are summarized in Table 1. 179 (42.2%) of 424 subjects had frailty. Mean age, DAS28-CRP, HAQ-DI, grip strength, and the proportions of those with finger/wrist joint symptoms, diabetes mellitus, hypertension, osteoporosis, and ever or current malignancy were all significantly different between those with and without frailty.

**Table 1:** Demographics and clinical characteristics of subjects.

Variables	Total (n = 424)	Non-frailty (n = 245)	Frailty (n = 179)	p value
Age (years), mean (SD)	66.8(14.5)	62.7 (14.5)	72.4 (12.7)	< 0.001
Duration of disease (years), mean (SD)	13.0 (10.6)	12.0 (10.1)	14.3 (11.2)	0.023
BMI (kg/m^2^), mean (SD)	21.6 (3.8)	21.4 (3.8)	21.8 (3.9)	0.280
Married (%)	61.3	66.5	54.2	0.012
Living alone (%)	17.0	16.3	17.9	0.696
Education ≥ 13 years (%)	30.0	34.3	24.0	0.024
Steinbrocker stage (3/4) (%)	40.8	35.8	47.5	0.020
Steinbrocker class (3/4) (%)	15.6	4.6	30.5	< 0.001
Glucocorticoid use (%)	31.4	24.9	40.2	0.001
Methotrexate use (%)	65.6	72.7	55.9	< 0.001
Other csDMARD use (%)	42.7	41.2	44.7	0.488
bDMARD or tsDMARD use (%)	39.2	37.1	41.9	0.365
Diabetes mellitus (%)	7.3	4.9	10.6	0.036
Hypertension (%)	18.4	12.2	26.8	< 0.001
Osteoporosis (%)	30.9	21.6	43.6	< 0.001
Interstitial pneumonia (%)	9.7	7.3	12.8	0.068
Ever or current malignancy (%)	7.8	5.3	11.2	0.029
Rheumatoid factor positive (%)	72.1	71.1	73.6	0.656
CRP (mg/dl), mean (SD)	0.4 (1.2)	0.3 (0.6)	0.6 (1.7)	0.011
MMP-3 (ng/ml), mean (SD)	86.2 (98.4)	78.3 (91.0)	97.1 (107.0)	0.056
Swollen 28-joint count, mean (SD)	0.8 (2.0)	0.7 (1.6)	0.9 (2.4)	0.178
Tender 28-joint count, mean (SD)	2.1 (3.8)	1.3 (2.3)	3.2 (5.1)	< 0.001
Subject's assessment of pain VAS (mm), mean (SD)	20.5 (23.5)	14.2 (18.9)	29.1 (26.3)	< 0.001
Subject's global assessment of disease activity VAS (mm), mean (SD)	20.8 (22.9)	14.4 (18.5)	29.6 (25.3)	< 0.001
Physician's global assessment of disease activity VAS (mm), mean (SD)	17.7 (20.4)	12.0 (15.8)	25.6 (23.2)	< 0.001
DAS28-CRP, mean (SD)	2.3 (1.0)	2.0 (0.9)	2.6 (1.1)	< 0.001
HAQ-DI, mean (SD)	0.54 (0.76)	0.20 (0.36)	1.01 (0.90)	< 0.001
KCL, mean (SD)	7.2 (4.8)	3.8 (2.0)	11.9 (3.5)	< 0.001
Dominant side (right/left/equal) (%)	45.1/35.6/19.3	47.8/34.3/18.0	41.3/37.4/21.2	0.401
Grip strength (kg), mean (SD)	17.4 (7.4)	19.7 (6.6)	14.1 (7.4)	< 0.001
Finger/wrist joint symptoms (%)	35.4	30.2	42.5	0.010

Frailty, The Kihon Checklist (KCL) ≥ 8 points.

*BMI* body mass index, *other csDMARD* conventional synthetic disease-modifying antirheumatic drug (DMARD) including salazosulfapyridine, tacrolimus, bucillamine, and iguratimod, *bDMARD* biological DMARD, *tsDMARD* targeted synthetic DMARD, *CRP* C-reactive protein, *MMP-3* matrix metalloproteinase-3, *VAS* visual analog scale, *DAS28-CRP* 28-joint count Disease Activity Score using C-reactive protein, *HAQ-DI* Health Assessment Questionnaire-Disability Index, *dominant side* side with the higher value of grip strength, *SD* standard deviation.

Table 2 shows significant regression coefficients of finger/wrist joint symptoms and grip strength for DAS28-CRP and HAQ-DI respectively. In ROC curves, cut-off scores of DAS28-CRP for finger/wrist joint symptoms were 2.2 (area under curve (AUC), 0.881; sensitivity, 82.7%; specificity, 76.9%), which corresponded to remission of disease activity^23^. Thus, we found that finger/wrist joint symptoms were significantly associated with DAS28-CRP, and grip strength was significantly associated with HAQ-DI.

**Table 2:** Linear regression analysis of DAS28-CRP and HAQ-DI.

Variables	Estimate (*B*)	(95% CI)	SE	*T*	p-value
**Contribution to DAS28-CRP**					
Age (years)	−0.03	(−0.08 to 0.02)	0.026	−1.01	0.313
Duration of disease (years)	−0.06	(−0.13 to 0.006)	0.035	−1.81	0.072
HAQ-DI	0.58	(0.48 to 0.68)	0.050	11.54	< 0.001
Finger/wrist joint symptoms	11.47	(10.01 to 12.94)	0.747	15.35	< 0.001
**Contribution to HAQ-DI**					
Age (years)	0.04	(−0.005 to 0.08)	0.021	1.73	0.084
Duration of disease (years)	0.07	(0.01 to 0.12)	0.028	2.35	0.019
DAS28-CRP	0.26	(0.20 to 0.31)	0.028	9.26	< 0.001
Grip strength (kg)	−0.43	(−0.52 to −0.34)	0.046	−9.36	< 0.001

P < 0.05 was considered statistically significant.

*DAS28-CRP* 28-joint count Disease Activity Score using C-reactive protein, *HAQ-DI* Health Assessment Questionnaire-Disability Index, *B* (regression coefficient) for every 0.1 point of HAQ-DI and DAS28-CRP, *SE* standard error, *T* t statistics.

Table 3 shows ORs for frailty based on multivariable logistic regression analyses. Since this study aimed to validate grip strength instead of HAQ-DI as a measure of frailty, we decided not to include HAQ-DI in the same model as grip strength. DAS28-CRP was entered as a variable in Model 1, whereas finger/wrist joint symptoms was entered as a variable in Model 2 (with comorbidities) and Model 3 (without comorbidities). Frailty was significantly associated with age, body mass index (BMI), DAS28-CRP (Model 1), finger/wrist joint symptoms (Model 3), and grip strength (Model 1/Model 2/Model 3).

**Table 3:** Odds ratios for frailty by logistic regression analyses.

Variables	Univariable OR			Multivariable OR (Model 1)			Multivariable OR (Model 2)			Multivariable OR (Model 3)		
	OR	(95% CI)	p-value	OR	(95% CI)	p-value	OR	(95% CI)	p-value	OR	(95% CI)	p-value
Age (years)	1.06	(1.04–1.08)	< 0.001	1.05	(1.03–1.07)	< 0.001	1.04	(1.02–1.06)	< 0.001	1.05	(1.03–1.07)	< 0.001
Duration of disease (years)	1.02	(1.00–1.04)	0.025		–			–			–	
BMI (kg/m^2^)	1.03	(0.98–1.08)	0.280	1.07	(1.00–1.14)	0.046	1.07	(1.01–1.14)	0.027	1.08	(1.01–1.14)	0.016
Married	0.60	(0.40–0.88)	0.010		–			–			–	
Education ≥ 13 years	0.61	(0.39–0.93)	0.023		–			–			–	
Steinbrocker stage (3/4)	1.62	(1.09–2.40)	0.017		–			–			–	
Diabetes mellitus	2.31	(1.09–4.88)	0.029		–			–				
Hypertension	2.63	(1.58–4.35)	< 0.001		–			–				
Osteoporosis	2.80	(1.83–4.28)	< 0.001		–			–				
Ever or current malignancy	2.24	(1.09–4.64)	0.029		–			–				
DAS28-CRP	1.91	(1.54–2.35)	< 0.001	1.74	(1.34–2.26)	< 0.001						
Finger/wrist joint symptoms	1.71	(1.14–2.55)	0.009					–		1.64	(1.01–2.65)	0.044
Grip strength (kg)	0.89	(0.86–0.92)	< 0.001	0.93	(0.89–0.97)	< 0.001	0.90	(0.87–0.94)	< 0.001	0.90	(0.86–0.94)	< 0.001

Frailty, The Kihon Checklist (KCL) ≥ 8 points.

*OR* odds ratio, *CI* confidence interval, *BMI* body mass index, *DAS28-CRP* 28-joint count Disease Activity Score using C-reactive protein.

**Figure 1:**
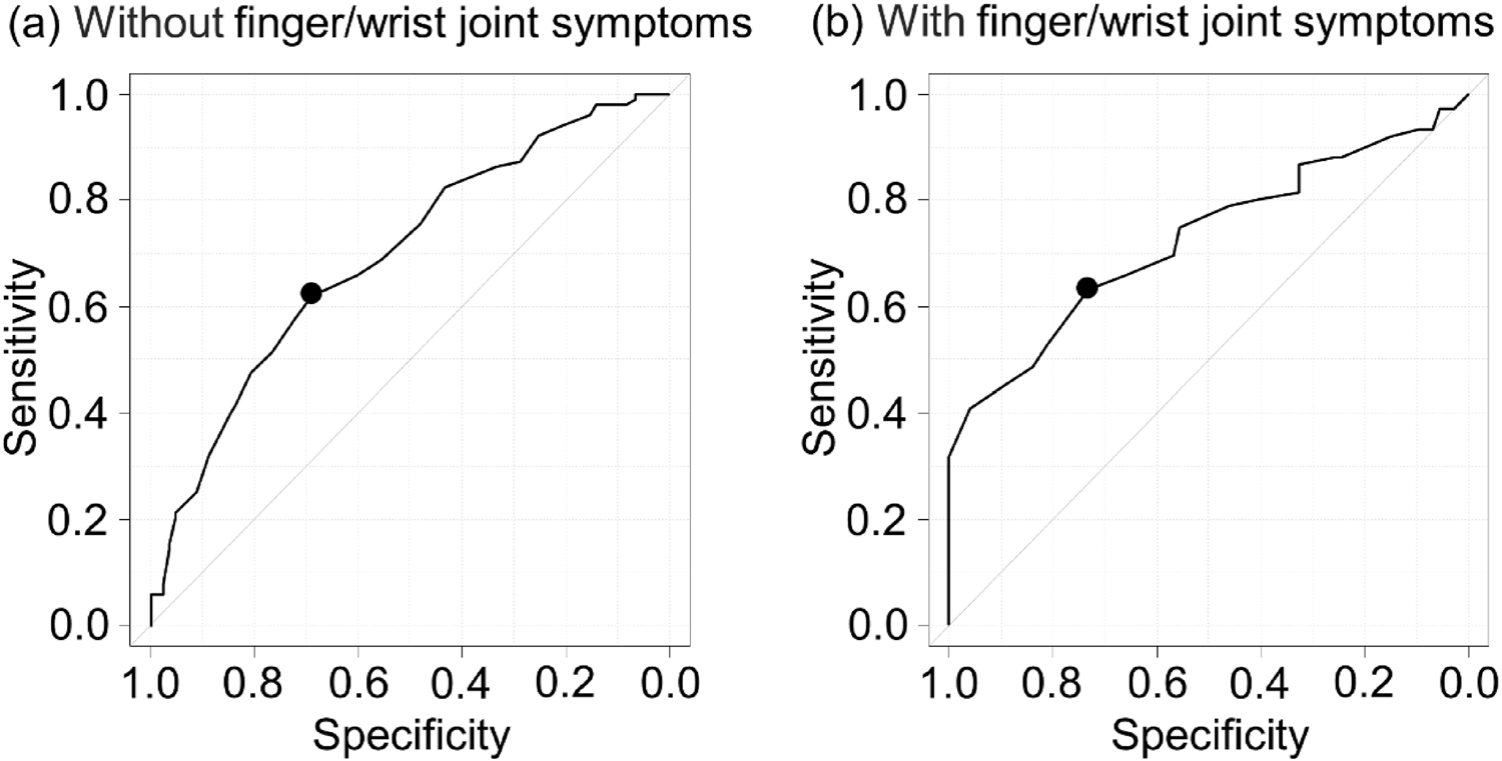
ROC curves for frailty and grip strength (**a**) without finger/wrist joint symptoms, and (**b**) with finger/wrist joint symptoms. Area under curve and Cut-offs were (**a**) 0.697 (95% CI: 0.633–0.761) and 17 kg (sensitivity, 62.1%; specificity, 69.0%), and (**b**) 0.726 (95% CI: 0.645–0.808) and 14 kg (sensitivity, 63.2%; specificity, 73.0%).

In the ROC curve analysis for frailty in subjects without finger/wrist joint symptoms, the best cut-off score of grip strength corresponding to frailty was 17 kg (sensitivity, 62.1%; specificity, 69.0%) (Fig. 1a). In subjects with finger/wrist joint symptoms, the best cut-off score of grip strength corresponding to frailty was 14 kg (sensitivity, 63.2%; specificity, 73.0%) (Fig. 1b).

**Figure 2:**
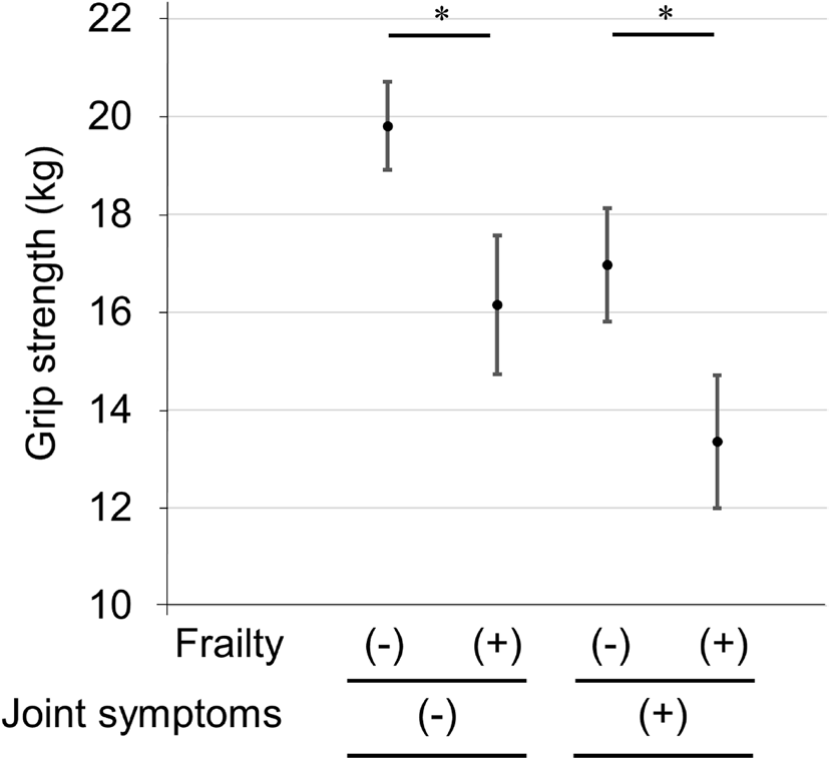
Estimated marginal means of grip strength with age, duration of disease, and BMI as covariates. In order from left to right, subjects with no frailty and no finger/wrist joint symptoms (n = 171, 40.3%), with frailty and no finger/wrist joint symptoms (n = 103, 24.3%), with no frailty and finger/wrist joint symptoms (n = 74, 17.5%), and with frailty and finger/wrist joint symptoms (n = 76, 17.9%).

Figure 2 shows estimated marginal means of grip strength adjusted for age, duration of disease, and BMI as covariates. Mean grip strength values were 19.8 kg (95% CI: 18.9–20.7) in subjects with no frailty and no finger/wrist joint symptoms, 16.2 kg (95% CI: 15.0–17.3) in subjects with frailty and no finger/wrist joint symptoms, 17.0 kg (95% CI: 15.6–18.4) in subjects with no frailty and finger/wrist joint symptoms, and 13.4 (95% CI: 12.0–14.7) in subjects with frailty and finger/wrist joint symptoms.

In ROC curve analysis, cut-off scores of grip strength for clinical remission (DAS28-CRP < 2.3)^23^/functional remission (HAQ-DI ≤ 0.5)^25^ in subjects without finger/wrist joint symptoms and with finger/wrist joint symptoms were 16 kg (sensitivity, 66.1%; specificity, 68.7%/sensitivity, 80.0%; specificity, 75.5%) and 14 kg (sensitivity, 51.7%; specificity, 76.5%/sensitivity, 69.6%; specificity, 75.3%), respectively, which were similar to cut-off scores of grip strength corresponding to frailty. When subjects were dichotomized according to CHS grip strength criteria to diagnose frailty (i.e., < 18 kg for females)^3,14^, the proportion of those with frailty was 58.5/26.7% (grip strength < 18 kg / ≥ 18 kg), the proportion of those with finger/wrist joint symptoms was 43.5/27.6%, and mean grip strength (SD) was 11.2 (4.5)/23.2 (4.4) kg, which were all significantly different between those with grip strength < 18 kg vs those with grip strength ≥ 18 kg.

## Discussion

The present study, to our knowledge, is the first attempt to investigate the association between grip strength and frailty in RA patients, with a specific focus on finger/wrist joint symptoms (i.e., tender and swollen joints) and disease activity (DAS28-CRP). Frailty was significantly associated with grip strength independently of age, comorbidities, and disease activity, which were previously reported to be factors related to frailty^27^, as well as finger/wrist joint symptoms. While the cut-off score of grip strength corresponding to frailty in RA patients without finger/wrist joint symptoms was 17 kg, which was similar to one of the CHS criteria for diagnosing frailty (i.e., < 18 kg for females)^3,14^, the cut-off score of grip strength in RA patients with finger/wrist joint symptoms was 14 kg, which was lower than that in those without finger/wrist joint symptoms. Furthermore, the upper limit of 95% CI for mean grip strength in subjects with frailty and no finger/wrist joint symptoms was 17.3 kg, and that for those with frailty and finger/wrist joint symptoms was 14.7 kg, indicating that RA patients with no finger/wrist joint symptoms having a grip strength less than 18 kg or RA patients with finger/wrist joint symptoms having a grip strength less than 15 kg are frailty. These findings suggest that grip strength in RA patients reflects frailty regardless of finger/wrist joint symptoms and disease activity. The cut-off score of grip strength corresponding to frailty is < 18 kg (equivalent to CHS criteria) when RA patients have no finger/wrist joint symptoms. However, when RA patients have finger/wrist joint symptoms, it may be considered to reduce the cut-off score of grip strength.

Finger/wrist joint symptoms were significantly associated with DAS28-CRP. In daily clinical practice, compared to the calculations of DAS28-CRP, information on finger/wrist joint symptoms can be obtained simply. We also found that grip strength was significantly associated with HAQ-DI. Based on the results of this study, we propose a screening method for evaluating frailty that uses grip strength in combination with finger/wrist joint symptoms. Actually, it is better to calculate DAS28-CRP and HAQ-DI to evaluate the condition of RA patients in detail. However, according to one report, grip strength of RA patients in remission of disease activity was almost equivalent to that of the healthy population^28^. Moreover, grip strength can be measured more easily than lower limb muscle strength and done in a shorter time than answering the questions of KCL, and finger/wrist joint symptoms can be identified immediately. Accordingly, it will be important to clarify which RA patients can be evaluated for frailty using grip strength, and when necessary, to lower the grip strength cut-off score when the patients have finger/wrist joint symptoms. The significance of our present findings is that it reveals the need to reduce the cut-off score of grip strength to predict frailty in RA patients with finger/wrist joint symptoms.

Cut-off scores of grip strength corresponding to clinical remission (DAS28-CRP < 2.3) and functional remission (HAQ-DI ≤ 0.5) were 16 kg (without finger/wrist joint symptoms) and 14 kg (with finger/wrist joint symptoms), respectively, which were similar to the cut-off score of grip strength corresponding to frailty. It was reported that frailty had significant association with disease activity^27^ and HAQ^29^. Notably, maintaining grip strength from the perspective of preventing frailty may lead to also aiming for clinical and functional remission, which can be the underlying goal of care in RA patients.

Factors that may influence grip strength in RA patients include age^30^ and disease activity^31^. In this study, even when adjusting for these factors, grip strength was significantly associated with frailty. Furthermore, reduced grip strength may also result from sarcopenia in RA patients^32^. While higher grip strength is reportedly associated with lower levels of inflammation, leading to lower mortality^33^, hand involvement in early inflammatory arthritis has been shown to be a strong predictor of a poor long-term disease outcome^34^. These results suggest that regular follow-up of grip strength and finger/wrist joint symptoms in RA patients may allow for the determination that disease activity is changing (worsening) and the prevention of frailty and the negative health outcomes. Adequate exercise can reduce pain in RA patients^35^ and improve grip strength^36^. From the perspective of preventing frailty, exercise may have a synergistic effect when performed in combination with RA drug therapy.

This study has several limitations. First, radiographic evaluations were not performed. However, we believe our findings are valid because tender and swollen joints could lead to reduced grip strength regardless of radiographic changes. Second, this study targeted only Japanese female RA patients. It will be important to conduct a study with a larger male RA patient population to confirm the cut-off score for males. Third, we did not obtain information regarding the history of upper limb surgery, which can affect grip strength^37^. Finally, physical factors of lower limb function such as walking time and psychosocial factors such as depression, anxiety, and social support, which are related to frailty^38^, were not evaluated in detail. Since some RA patients do not have frailty despite having poor grip strength, evaluating frailty based only on upper limb function is insufficient. Nonetheless, upper limb function measurements are simple to perform and may serve as a screening index for evaluating frailty in daily clinical practice.

In conclusion, we investigated the association between grip strength and frailty in RA patients. Frailty was significantly associated with grip strength, independently of age, disease activity, and finger/wrist joint symptoms. Measuring grip strength and checking finger/wrist joint symptoms offer a useful and simple way to assess frailty in daily clinical practice. Our findings serve as a foundation for the development of methods to detect and screen for frailty, as well as interventions.

## Data Availability

The datasets used and/or analysed during the current study available from the corresponding author on reasonable request.
